# Trait emotional intelligence mediates the dispositional gratitude and subjective well-being in young adults

**DOI:** 10.3389/fpsyg.2024.1414867

**Published:** 2024-08-08

**Authors:** Cristina Torrelles-Nadal, Agnès Ros-Morente, Carla Quesada-Pallarès

**Affiliations:** ^1^INEFC, National Institute of Physical Education of Catalonia, University of Lleida, Lleida, Spain; ^2^Pedagogy Department, Universitat de Lleida, Lleida, Spain; ^3^Applied Pedagogy Department, Autonomous University of Barcelona, Barcelona, Spain; ^4^Serra Húnter Fellow, Catalonia, Spain

**Keywords:** dispositional gratitude, trait emotional intelligence, life satisfaction, happiness, subjective well-being

## Abstract

Gratitude has proved to be an enhancer of subjective well-being in previous studies. However, studies that linked the relation between emotional intelligence and its facets when interacting with gratitude, are still limited. In this sense, this study examined the mediating roles of emotional intelligence between gratitude and subjective well-being indicators, by introducing the general factor of emotional intelligence. The first approach to data analysis was to examine the descriptive statistics; the second approach consisted of an Exploratory Structural Equation Modelling, applying also a bifactor analysis. Data was collected from 406 Spanish students, through an online survey that includes the gratitude, trait meta mood scale, satisfaction with life and subjective happiness scale. The mean age of participants was 20.27 years (*SD* = 4.68), whereas 79.5% were females. The results provided preliminary evidence of the mediation role of the general factor of emotional intelligence between gratitude and subjective well-being, which provided a meaningful insight about the role of trait emotional intelligence. These findings suggested that gratitude promotes emotional intelligence, allowing to an increase in subjective well-being. Nonetheless, there is a need for further research to achieve a better understanding of the role of the emotional intelligence facets between gratitude and subjective well-being.

## 1 Introduction

Recently, the positive psychology field has acknowledged the benefits of gratitude and it has become a key construct in psychology research in order to understand social and emotional development of individuals (Bausert et al., 2018). Research has found evidence for a relationship between gratitude and a wide range of measures of well-being, showing that grateful people tend to experience a higher degree of life satisfaction and positive affect than those who have lower levels of gratitude (Xiang and Yuan, 2020). Additionally, having high levels of gratitude is also related to scoring higher on measures of prosocial behavior, empathy, self-esteem, and state of gratitude (McCullough et al., 2002; Kong et al., 2015; Jackowska et al., 2016; Shi and Du, 2020). Additionally, research shows that those individuals who report higher feelings of gratitude appear as more likely to report lower levels of symptomatology linked to negative emotions, such as depression, anxiety, envy or perceived stress (McCullough et al., 2002; Lee et al., 2018).

Given this evidence, and as an attempt to explain these relationships between gratitude and its benefits, researchers have pointed out the possibility that gratitude helps to reinforce those personal resources, as physical resources (e.g., health longevity); social resources (e.g., social support, loyalty), intellectual resources (e.g., expert knowledge) and psychological resources (e.g., resilience, optimism, and creativity) (Fredrickson et al., 2003). This reinforcement is carried out thanks to the ability of positive emotions to be able to expand the momentary repertoire of thought and action that the individual has, and could constitute one of the underlying factors that explain well-being and mental health (Fredrickson et al., 2003; Fredrickson and Branigan, 2005; Froh et al., 2009; Killen and Macaskill, 2015). For example, according to Fredrickson’s (2013) broaden-and-build theory, gratitude can help building and enduring personal resources, as well as enhance emotional intelligence and subjective well-being.

Actually, gratitude could work in a similar way to emotional intelligence. Decades of study have revealed the role of emotional intelligence effects over various relevant variables including general health, socio-emotional outcomes, and life satisfaction (Charoensukmongkol, 2014; Jacobs et al., 2016). Despite the extensive research on gratitude and emotional intelligence separately, scarce studies (Geng, 2018; Chang et al., 2022) investigated the relationship of these different constructs with subjective well-being (SWB).

Because of all the described above, this study focuses on testing whether general emotional intelligence and its emotional facets (mood attention, emotional clarity and mood repair) could mediate the relationship between gratitude and SWB indicators (i.e., life satisfaction and happiness).

### 1.1 Gratitude

From the approach of positive psychology, gratitude is conceptualized as “an emotion, an attitude, a moral virtue, a habit, a personality trait, and a coping response” (Emmons et al., 2003, p.327; Krause, 2006, p.164). According to the large volume of literature, emotional well-being, mental health and physical health are influenced by the trait of gratitude, which has an important role in them (Fredrickson, 2013; Kong et al., 2020). Hence, the present study focused on the trait of gratitude.

Wood, et al. (2010, p.1) define gratitude as a trait like a “part of a wider life orientation towards noticing and appreciating the positive in the world.” Thus, individuals with high levels of trait gratitude tend to experience and express gratitude more frequently and with greater intensity than those with lowers levels of trait of gratitude. This, at the same time, has a direct relation with their thoughts and actions (McCullough et al., 2004; Gallagher and Vella-Brodrick, 2008; Watkins et al., 2014).

Grateful people seem to show positive associations with more frequent and intense experiences of gratitude states in daily life, empathy, life satisfaction, well-being, enhance emotional well-being and a higher number of positive emotions compared to those with lower scores (McCullough et al., 2004; Watkins et al., 2014; Kong et al., 2015).

Gratitude could be considered as a life orientation, that helps and facilitates people focusing and appreciating the bright side of life. In other words, higher levels of gratitude can increase the good in one’s life (Wood et al., 2010; Watkins et al., 2014), and this attitude can be displayed as a personality trait (Lin and Yeh, 2014a; Xiang and Yuan, 2020).

Grateful people are more open-minded and flexible, and have the capacity to see and take advantage of more opportunities in the environment (Fredrickson, 2004; Fredrickson and Branigan, 2005; Johnson and Fredrickson, 2005; Watkins et al., 2019). Moreover, grateful people have the capacity to find benefits in difficult circumstances, which could be considered an effective coping mechanism (Lin and Yeh, 2014b) and an adaptive psychological strategy (Salvador-Ferrer, 2017). In this sense, according to authors such as Valikhani et al. (2019) there is multiple evidence that gratitude is inversely associated with anxiety, depression, or negative affect. This is possibly explained by the fact that gratitude is conceived as focusing on the positive aspects of one’s life, becoming the opposite of negative affect variables and disorders. People expressing gratitude have the capacity to widen their focus of attention and cognition, facilitating them to increase their personal resources to help them to achieve better lives in the future. This is related to the broaden-and-build theory of positive emotions (Fredrickson, 2013). According to the author, gratitude helps people to build and increase resources that allow them to improve their well-being and allows them to adapt in unpleasant circumstances (Conway et al., 2013). Dispositional gratitude allows people to protect themselves from a variety of mental disorders (e.g., depressive symptoms, hypochondriasis, suicide) (Lyubomirsky et al., 2005) and has the capacity to increases their life satisfaction and happiness (Lyubomirsky et al., 2005; Froh et al., 2009; Lambert et al., 2012; Kong et al., 2015, 2020; Watkins et al., 2019). Thus, grateful people have the facility to clearly recognize all the things and people that are significant and necessary to their well-being (Fredrickson, 2013).

### 1.2 Gratitude, emotional intelligence and subjective well-being

#### 1.2.1 Gratitude and emotional intelligence

Salovey and Mayer (1990) defined Emotional Intelligence (EI) as a set of abilities that allow people to process emotional information and perceive, use, understand, and manage emotion to guide their thinking and actions for an optimal adaptation and promote emotional and intellectual growth. Moreover, people obtain information from their emotions which may play an important role in promoting personal growth (Rey and Extremera, 2014). However, even nowadays, there is not an agreement among researchers about the existence and conceptualization of EI. The most extended theoretical approaches are trait EI and ability EI (Blasco-Belled et al., 2019; O’Connor et al., 2019; Tejada-Gallardo et al., 2020). According to Petrides (2011) and Petrides et al. (2016), trait EI is understood as people’s self-perception of their emotional ability to allow people to guide in the process of adaptive coping and maintaining an adequate level of well-being. In fact, trait EI explains the self-perceptions of our emotional world (Petrides et al., 2018). In contrast, Mayer et al. (2016) understand ability EI as the cognitive-emotional ability that people have to perceive, use, understand and regulate emotions.

Scholars showed that trait EI is highly related to positive outcomes as healthy adaptation (Szczygieł and Mikolajczak, 2017; Tejada-Gallardo et al., 2020), and well-being (Di Fabio and Kenny, 2016). Chang et al. (2022) analyzed the link between gratitude and satisfaction with life and the role of trait EI. Furthermore, different meta-analyses have demonstrated greater significance for trait EI on health and life satisfaction compared to ability (Martins et al., 2010; Sánchez-Álvarez et al., 2016; Moroń and Biolik-Moroń, 2021). According to Schutte et al. (2021, p.2614) “trait emotional intelligence tended to have higher associations with outcomes than ability emotional intelligence.” In addition, trait scales are more appropriate than ability scales because the former tend to have very good psychometric properties, generally higher the internal consistency, better factor structures, and a solid theoretical basis (Extremera et al., 2007; Siegling et al., 2015; O’Connor et al., 2019; Shi and Du, 2020; Tejada-Gallardo et al., 2020). According to this, within the present study, we evaluated self-perceived EI, therefore, we used a trait EI self-report to capture general EI. Trait Meta-Mood Scale (TMMS; Salovey et al., 1995), is one of the most used self-report measures of EI in Spain and Latin America (Martínez-Monteagudo et al., 2019), which tackles the meta-knowledge of emotional states scale. It enables people to become aware of their own emotional skills, and considers three main facets: attending to one’s own and others’ affective responses (mood attention), identifying and understanding these emotional patterns (emotional clarity), and being able to regulate one’s own and others’ emotions so as to cope with situational demands (mood repair).

Scholars show the relationship between emotional intelligence and SWB. Indeed, the role between emotional intelligence facets (emotional clarity and mood repair) and SWB (Extremera et al., 2011; Prado-Gascó et al., 2018; Blasco-Belled et al., 2019; Martínez-Marín and Martínez, 2019). According to Sánchez-Álvarez et al. (2016), despite there is a positive relationship between EI and SWB components and a stronger overall relationship in trait-EI studies, sometimes it is possible to find some controversial findings. Scholars showed that EI had better association with the cognitive component of SWB (satisfaction with life) than the affective component of SWB (happiness or PANAS). According to Blasco-Belled et al. (2019, p.2043) “it seems that high levels of EI result in greater SWB.” Taking into consideration the EI facets, the scholars showed that emotional clarity is a positive predictor of life satisfaction, whereas mood repair has a positive relationship with life satisfaction (cognitive component of SWB). One of the reasons could be that trait-EI have a temporal stability (it does not vary according to time) and life satisfaction is considered a global index in time; on the other hand, happiness, the affective component of SWB, is most moldeable in time (Extremera et al., 2011; Prado-Gascó et al., 2018; Martínez-Marín and Martínez, 2019; Tejada-Gallardo et al., 2020). However, regarding the mood attention facet, studies show that this facet was associated with to lower levels of satisfaction with life (Extremera et al., 2011) and happiness (Blasco-Belled et al., 2019). Even though the vast majority of the literature analyses the relation between emotional clarity and mood repair and SWB indicators, it is true that the relation between mood attention and SWB indicators is still needs to be explored in depth. According to Sánchez-Álvarez et al. (2016) global EI and its facets are considered more effective predictors of the cognitive component of SWB (life satisfaction) than the affective component (happiness).

Few studies have analyzed the relationship between EI and gratitude (Anming et al., 2012; Rey and Extremera, 2014; Geng, 2018; Chang et al., 2022). The different reports show the positive correlations between both, and EI can significantly predict gratitude. Szcześniak et al. (2020) state that emotional intelligence enhances gratitude and vice versa; in other words, their relationship could be considered as an upward spiral, with both variables having the capacity to feedback on each other. Salve and Lavalekar (2017) showed that gratitude is a good predictor of EI, and according to them, other scholars (McCullough et al., 2004; Fredrickson, 2013; Watkins et al., 2014; Kong et al., 2015) consider that being grateful enhances the EI. Fredrickson (2004), following the theory of the broaden-and-build model -in which positive emotions have the capacity to expand the momentary repertoire of individual thought-action and allow to increase personal resources-, it is possible to explain that gratitude has the capacity to expand and raise EI.

McCullough et al. (2002) considered the trait of gratitude as a gratitude disposition to allow people to recognize and respond in a positive emotion to people, acting in a benevolence way. Grateful people have the capacity to improve their perspective on life, it allowing them to accurately identify, understand and modify their emotional states. Watkins et al. (2001) showed a significant relationship between dispositional gratitude and trait-EI (general *e*-factor and its three facets). Therefore, trait gratitude might promote EI, and “predict better emotional well-being than other important personality traits” (Watkins, 2014, p. 57).

Therefore, gratitude seems to be a factor which, together with other factors that mediate, can bring the individual to a healthier or protected outcome and a better sense of subjective well-being. In addition, gratitude has proved to be related to satisfaction with life, which are important predictors of happiness. However, some authors claim that more studies should be carried out in order to explore in further detail the mediating roles of various psychological constructs that show an association to gratitude, and analyze their relationship with well-being (Wood et al., 2010; Watkins, 2014). One of them could be trait EI, which was associated with positive outcomes as positive affect or life satisfaction (Park and Dhandra, 2017). Furthermore, the mediating effect of trait EI has been explored in different studies (Watkins et al., 2001; Johnson et al., 2009; Park and Dhandra, 2017; El-Khodary and Samara, 2019; Schutte et al., 2021; Chang et al., 2022). It is interesting to note that the mediating role of EI can be observed in each of the studies. Therefore, the current study aims to investigate the mediating role of general EI and its three main facets in the relationship between dispositional gratitude and SWB indicators.

#### 1.2.2 The e-factor model in the study of EI

Despite the existence of some literature showing that EI cannot be considered as a unidimensional construct (Mayer et al., 2016; Delhom et al., 2017), there are other literatures and researches analyzing that it is (Koydemir et al., 2012; Gutiérrez-Cobo et al., 2017; Szczygieł and Mikolajczak, 2017). Blasco-Belled et al. (2019) tried to explain which were the implications of the *e-*factor EI model, and to that they compared the structural organization of EI with general intelligence. Whereas intelligence is explained by the *g*(eneral)-factor of intelligence, that represents the self-perceptions regarding the ability of people to deal with emotions can be explained by the *e*(motional)-factor of EI. Moreover, it is possible to describe each independent ability of EI throughout the specific dimensions of mood attention, emotional clarity, and mood repair (compared with intelligence’s specific dimensions) (Tejada-Gallardo et al., 2020). The *e*-factor EI model stands for the general EI construct as a bifactor model, which contains information of all the items and takes into consideration the contribution of each facet of EI (mood attention, emotional clarity and mood repair) to the outcomes of interest. Indeed, the *e*-factor EI model as a bifactor -a multidimensional model- is used both as a Confirmatory Factor Analysis and ESEM (Exploratory Structural Equation Modelling), as other authors suggest in order to provide an alternative to hierarchical models (Morin et al., 2016). Despite existing different studies which used the *e-*factor model to associate with SWB (Blasco-Belled et al., 2019; Tejada-Gallardo et al., 2020), this structure has not been used with gratitude yet.

#### 1.2.3 Influences of gratitude on subjective well-being

Subjective well-being refers to the appraisal that people make regarding their lives and the emotional experiences they have (Diener et al., 2015). According to Diener et al. (1999), this construct has cognitive and affective components, which define its complex nature. The cognitive component is defined by a general and global assessment of the life of the individual and it could be understood as Life Satisfaction. In contrast, the more affective component is characterized by the positive emotional experiences of the individual (positive affect), is also regarded as happiness (Diener et al., 2003; Strobel et al., 2011; Diener et al., 2012; Szczygieł and Mikolajczak, 2017; Blasco-Belled et al., 2019). According to Szczygieł and Mikolajczak (2017) satisfaction with life and subjective happiness are the components most used to evaluate SWB.

SWB is considered a highly important factor for the adjustment of individuals, and improving SWB can effectively enhance the mental health of an individual (Geng, 2018). In fact, it is important to note that life events and circumstances play a role in determining and modifying subjective well-being, together with the management of emotions. Thus, the subjective appraisals of these experiences could be viewed as an indicator of whether people are leading a well-lived life (Diener et al., 2018).

Scholars have demonstrated that gratitude has positive correlation and is causally involved in the improvement of subjective well-being; also, grateful people tend to experience a high degree of life satisfaction and positive affect, as well as empathy (Emmons and Crumpler, 2000; McCullough et al., 2002; Kong et al., 2015; Boggio et al., 2019). Dickens (2017) conducted a metanalysis showing that gratitude interventions have positive effects on well-being, happiness, and life satisfaction.

As suggested by some authors, all these variables, however, should be included in a perspective of positive intervention which leads to a better managing of emotions, taking into account possible underlying processes, which lead to better outcomes, such as happiness and well-being (Quoidbach et al., 2015; Witvliet et al., 2019).

Watkins et al. (2014) demonstrated that grateful people are relatively active with positive affects, such as satisfaction, happiness and hope and they experience fewer negative emotions. Moreover, Watkins et al. (2014) and Unanue et al. (2019) indicate the potentiality of gratitude and SWB, and both can strengthen each other in a virtuous circle of events (e.g., the virtuous circle is created through acts of gratitude produced by people. When somebody produces or generates an act of gratitude, this can generate a spiral effect, when one act of gratitude is realized, another is generated and so on progressively until the virtuous circle is created).

Studies showed that gratitude can significantly predict SWB (Kong et al., 2015; Chaves et al., 2016; Jackowska et al., 2016), and demonstrated that it is important to human happiness (Watkins et al., 2014).

However, there are some studies have shown the mechanism by which gratitude modulates well-being. In fact, Boggio et al. (2019) and Chang et al. (2022) demonstrated emotion regulation as one of these mechanisms. Therefore, it is necessary to analyze if gratitude effects on subjective well-being are explained by its effects on general EI (*e*-factor) and its facets, and according to Foji et al. (2020), it is necessary to explore the roles of the different emotional intelligence’s facets on subjective well-being, and their role as mediators between gratitude and subjective well-being.

## 2 The current study

In short, the purpose of this study was to explore the relations underlying the association between gratitude and subjective well-being indicators, i.e., life satisfaction and happiness. Moreover, according to the theoretical rationale developed above, the aim was to: disentangling the contribution of emotional intelligence facets (mood attention, emotional clarity and mood repair), exploring the general EI (*e*-factor) as a potential mediator between gratitude and SWB indicators, and identifying the different roles in consideration of cognitive and affective components of SWB. Therefore, the following hypotheses were proposed: (H1) the model that better explains the relationship between gratitude and SWB is the one that includes both the general EI (*e*-factor) and EI facets; (H2) there is a positive relation between gratitude and SWB; and (H3) the *e*-factor EI (or the EI facets) mediate between gratitude and SWB.

**Figure 1:**
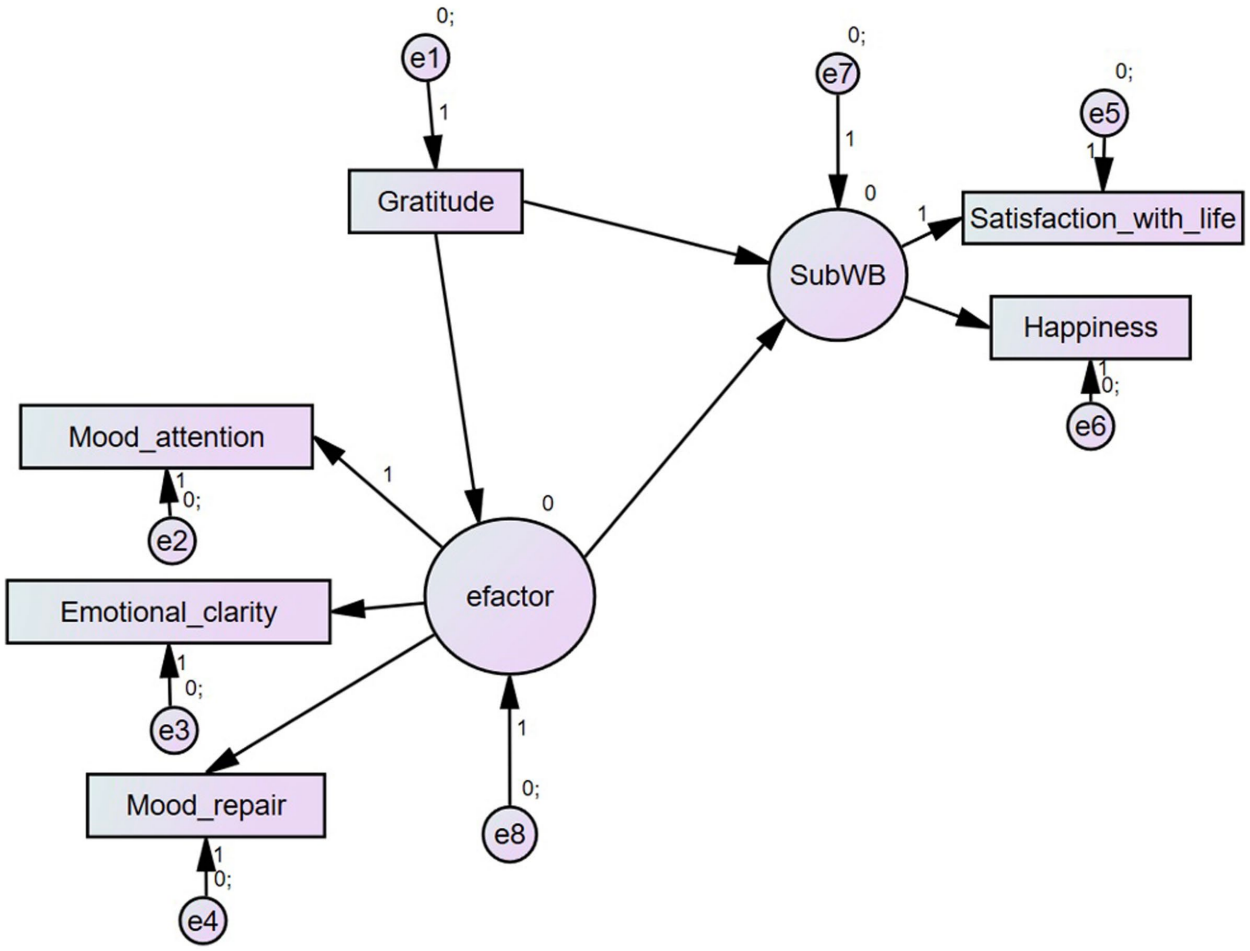
Hypothetical model A. Gratitude on Subjective Well-being (SubWB or SWB) as a factor of Satisfaction with life and Happiness, using TMMS facets from e-factor as a mediator.

Based on these aims, hypotheses and the literature, we formulated our hypothetical model, which assumed structural and causal relationship among gratitude and SWB, adding EI is a mediator between gratitude and SWB indicators (see Figure 1).

## 3 Methods

### 3.1 Participants and procedure

A cross-sectional study of university students was conducted. Specifically, a total of *N* = 406 first-year students (79.5% female) between 17 and 59 years old (*M* = 20.27; *SD* = 4.68) completed an on-line questionnaire. According to different scholars (Ding et al., 1995; Kline, 2016), our sample size -which exceeds 200- was an adequate sample. A non-probabilistic convenience sampling technique was used.

The participants enrolled voluntarily, and they could withdraw from the study at any time without further explanation; no partial responses were collected. No compensation was given for participating in the study. An informed consent was attached to the questionnaire, which explained the aims and voluntariness of the study. Once they signed the informed consent, the participants were able to start the questionnaire. No missing observations were recorded because responses were collected online and each question was mandatory.

### 3.2 Measures

Below, the instruments selected and applied in our study are presented, including reliability measures, which can be found in Table 1, and the results of their confirmatory factor analysis.

**Table 1:** Descriptive statistics, reliability estimates and Pearson’s coefficients of the variables used (*N* = 406).

Measures	Scale	M (SD)	Kurtosis	Skew	α	1	2	3	4	5	6	7	8
GQ-6	(1) Gratitude	4.91 (0.59)	0.445	−0.160	0.74	–							
TMMS-24	(2) *e*-factor (general EI)	3.58 (0.55)	1.06	−0.45	0.90	0.234^**^	–						
	(3) Mood attention	3.78 (0.73)	0.006	−0.437	0.88	0.093	0.670^**^	–					
	(4) Emotional clarity	3.44 (0.77)	−0.323	−0.161	0.89	0.208^**^	0.782^**^	0.273^**^	–				
	(5) Mood repair	3.53 (0.74)	0.015	−0.191	0.85	0.212^**^	0.747 ^**^	0.426^**^	0.426^**^	–			
SWB	(6) Subjective well-being	4.75 (0.99)	0.18	−0.450	0.86	0.280^**^	0.399^**^	−0.043	0.481^**^	0.427^**^	–		
	(7) Happiness	4.85 (1.02)	0.062	−0.323	0.70	0.227^**^	0.354^**^	−0.105^*^	0.4458^**^	0.4277^**^	0.354^**^	–	
	(8) Life satisfaction	4.66 (1.18)	0.079	−0.443	0.84	0.276^**^	0.368^**^	0.019	0.427^**^	0.353^**^	0.368^**^	0.639^**^	–

M (Mean); SD (Standard Deviation); GQ-6: gratitude scale; TMMS-24: Trait meta-mood scale; SWB: Subjective well-being construct; SHS: Subjective happiness scale; SWLS: Satisfaction with life scale; ^**^Correlation is statically significant at 0.01 (2-tails); ^*^Correlation is statically significant at 0.05 (2-tails).

Gratitude Questionnaire (GQ-6) is a 6-item scale measuring levels of dispositional gratitude (McCullough et al., 2002, Spanish version: Langer et al., 2016); Scale responses range from 1 (*strongly agree*) to 7 (*strongly disagree*). A sample item is ‘*I have so much in life to be thankful for.*’ Higher scores indicate greater dispositional gratitude. Previous research indicated good internal consistency with *α* of 0.82 (McCullough et al., 2002). The present study found similar internal consistency with Cronbach’s *α* = 0.74. Moreover, confirmatory factor analysis indicated that the GQ-6 had good construct validity (*χ2* (N = 406) = 642.021, *df* = 15, *p* < 0.001, *CFI* = 0.98, *TLI* = 0.97, *RMSAE* = 0.06 [0.025–0.093]; *SRMR* = 0.032; *BIC* = 7492.484).

Trait Meta-Mood Scale (TMMS-24; Salovey et al., 1995; Spanish version: Fernandez-Berrocal and Ramos, 2004). This instrument comprises 24 items assessing perceived EI that provides scores for three dimensions or facets: mood attention, emotional clarity, and mood repair. It uses a 5-point Likert scale (1 = strongly disagree to 5 = strongly agree). A sample item for each of the facets is: ‘*I think about my mood constantly*’ (mood attention), ‘*I am usually very clear about my feelings*’ (emotional clarity), and ‘*Although I am sometimes sad, I have a mostly optimistic outlook*’ (mood repair). The reliability for *e-*factor EI is 0.90 and for each dimension is 0.88, 0.89, and 0.85, respectively. Moreover, confirmatory factor analysis indicated that the TMMS-24 had good construct validity (*χ2* (N = 406) = 4878.911, *df* = 276, *p* < 0.001, *CFI* = 0.94, *TLI* = 0.93, *RMSAE* = 0.054 [0.048–0.061]; *SRMR* = 0.062; *BIC* = 23219.910).

Satisfaction with Life Scale (SWLS; Diener et al., 1985; Spanish version: Atienza et al., 2000). This is a 5-item questionnaire that evaluated the degree of satisfaction with life as a whole. Participants are asked to rate their satisfaction with life on a 7-point Likert-scale (*1 = strongly disagree* to *7 = strongly agree*). A sample item is ‘*So far I have gotten the important things I want in life’.* The reliability estimate was good, α = 0.84. Moreover, confirmatory factor analysis indicated that Satisfaction with life had good construct validity (*χ2* (N = 406) = 425.557, *df* = 6, *p* < 0.001, *CFI* = 0.99, *TLI* = 0.98, *RMSAE* = 0.045 [0.00–0.0117]; *SRMR* = 0.016; *BIC* = 5267.278).

The Subjective Happiness Scale (SHS; Lyubomirsky and Lepper, 1999; Spanish validation: Extremera and Fernández-Berrocal, 2014) assessed the level of one’s individual happiness (‘*In general, I consider myself…’*). It comprised 4 items (one reversely-scored) in which participants assessed their response using a 7-point Likert scale (with different response options for each item; e.g., 1 = *not a very happy person* to 7 = *a very happy person*). Within the current study, the reliability estimate was acceptable, *α* = 0.70. Moreover, confirmatory factor analysis indicated that the subjective happiness had good construct validity (*χ2* (N = 406) = 660.461, *df* = 1, *p* < 0.001, *CFI* = 0.98, *TLI* = 0.97, *RMSAE* = 0.06 [0.019–0.104]; *SRMR* = 0.024; *BIC* = 6588.850).

### 3.3 Statistical analysis

Testing for normality was the first approach to analysing the data. The skewness and kurtosis values were between −2 and + 2, which was considered acceptable in order to prove normal univariate distribution (George and Mallery, 2010), indicating similarity to the normal curve.

Then, we conducted descriptive and correlational analyses (see Table 1, along with reliability measures).

The measurement model included 2 s-order factors such as the *e*-factor -composed by mood attention, emotional clarity, and mood repair- as well as the subjective well-being -composed by happiness and life satisfaction-. This was the hypothesized model we named A (see Figure 1). In Table 1 we also provided the alpha values for both factors, which were higher than their first-order factors.

Model A included 6 observed variables (mood attention, emotional clarity, mood repair, gratitude, happiness, and life satisfaction) and 2 latent variables (e-factor and subjective well-being). Using this model, we later examined whether EI mediated the relationship between dispositional gratitude and satisfaction with life and happiness.

Towards this end, the two-step procedure recommended by Anderson and Gerbing (1988) was adopted to analyze the mediation effects. First step consisted of a test measurement model to confirm that each of the latent variables was represented by its indicators. And the second step allowed us to examine the structural model and determine if the measurement model was a good fit. The subsequent analyses used IBM AMOS Graphics v.23 to conduct Exploratory Structural Equation Modelling (ESEM); (Asparouhov and Muthén, 2009). ESEM is a procedure that dovetails elements of exploratory and confirmatory factor analyses; ESEM allows for the computation of statistics evaluating goodness of fit for the model. In the assessment of the tested model, different indices and their suggested cut-offs were relied upon (Kula and Guler, 2014): Chi-square with a non-significant *p*-value (Wan, 2002); Root Mean Square Error of Approximation (RMSEA) with values equal to or below 0.06 as evidence of a well-fitting model (Hu and Bentler, 1999); Comparative Fit Index (CFI), and Tucker-Lewis Index (TLI), both requiring values above 0.90 for acceptable data-model fit (Byrne, 1994; Kline, 2016); and Hoelter’s critical N (or Hoelter index), which informs if the sample size in the study is acceptable (above 75) or good (above 200) to carry out the analyses intended. The maximum likelihood method (MLM) was used. Finally, the direct and indirect effects of the relationships established among the variables were analyzed. The bootstrap technique was used to determine significance, as suggested by Collier (2020) when using mediation testing.

All of the data necessary to replicate the results are available in an open repository at: https://osf.io/zgbrp/?view_only=53e9b3bd48624caf86cf1956a800dd6f.

## 4 Results

### 4.1 Descriptive, reliability estimates and correlations

Table 1 showed the results obtained from the descriptive statistics, reliability estimates of the measured variables: *e*-factor, the three EI facets, gratitude, subjective well-being, life satisfaction and happiness. All scales showed good internal consistency (values ≥0.70). Furthermore, there were significant correlations (Pearson’s coefficients) among all the factors considered in the tested model, except mood attention with gratitude, subjective well-being, and life satisfaction. Also, all correlations were positive except for mood attention and happiness.

### 4.2 Measurement model

As planned in our hypothetical model A, we tested whether the model had good fit according to fit indexes. Model A suggested that the EI facets are grouped by a second-order factor (*e*-factor), which mediates between gratitude and subjective well-being (a second-order factor that includes happiness and satisfaction with life). Table 2 provided with all the results, in which we basically observed that chi-square was significant, CFI and TLI had values below 0.90, and RMSEA obtained values above 0.06; however, Hoelter’s value of 81 informed us that the sample size was adequate, at least. Considering that the measurement model A did not present a good fit, and following the latest advances in the theory of gratitude and emotional intelligence, we created two more models.

**Table 2:** Fit indices of the tested models.

Model	*N*	*X*^2^ (df), *p*	CFI	TLI	AIC	RMSEA [90% CI]	Hoelter (*p* < 0.05)
Model A: Gratitude on SWB, with e-factor (as a latent variable) and TMMS facets (as observed variables), as mediators	406	70.890(7), *p < 0*.001	0.883	0.749	110.890	0.150 [0.120–0.183]	81
Model B: Gratitude on SWB, with *e-*factor (as an observed variable) as a mediator	406	0.752(1), *p = 0*.386	1.000	1.005	26.752	0.000 [0.000–0.125]	2069
Model C: Gratitude on SWB, with TMMS scales (as observed variables) as mediators	406	65.524(4), *p < 0*.001	0.887	0.577	111.524	0.195 [0.155–0.238]	59
Model D: Gratitude on SWB, with e-factor and TMMS facets (as latent variables), as mediators. All items included	406	0.000(0), n.s.	1.000	–	1638.000	0.166 [0.163–0.169]	37

Model B simplified the previous model and used only the *e*-factor as an observed variable, not including the EI factors separately. In this case, the *e*-factor mediated between gratitude and subjective well-being (as a second-order factor). Fit indices, informed us of a non-significant chi-square value, as Table 2 showed, CFI and TLI values above 0.95, a RMSEA value below 0.06, and a high Hoelter’s value indicating a good sample size.

We also tested Model C to ensure that including the *e*-factor was the best choice. In this case, we used the EI facets as observed variables, without the *e*-factor, acting as mediators between gratitude and subjective well-being (as a second-order factor). Fit indices suggested a bad fit of the model: model C obtained a significant chi-square value, CFI and TLI values below 0.90, a RMSEA value above 0.06 and a Hoelter’s value below 70, which indicated that the sample size was not enough to test the specific model.

Finally, we tested Model D as the most complex one because we also included all the items of the scales. Indeed, it was a complex version of Model A. Fit indices suggested a poor fit of the model: model D got no chi-square p-value, which indicated a problem with the model; a CFI below 0.90, no TLI value, a RMSEA value above 0.06, and a Hoelter’s value below 70, which again indicated that the sample size was not enough to test the specific model. In fact, not obtaining some of the data suggested that the complexity of the model is not well-computed with the sample collected.

Considering all the information from the measurement models, we decided to accept Model B as the one with better fit. This outcome refuted our first hypothesis (H1) in which we assumed that model A would obtain the best fit.

### 4.3 Structural model

**Figure 2:**
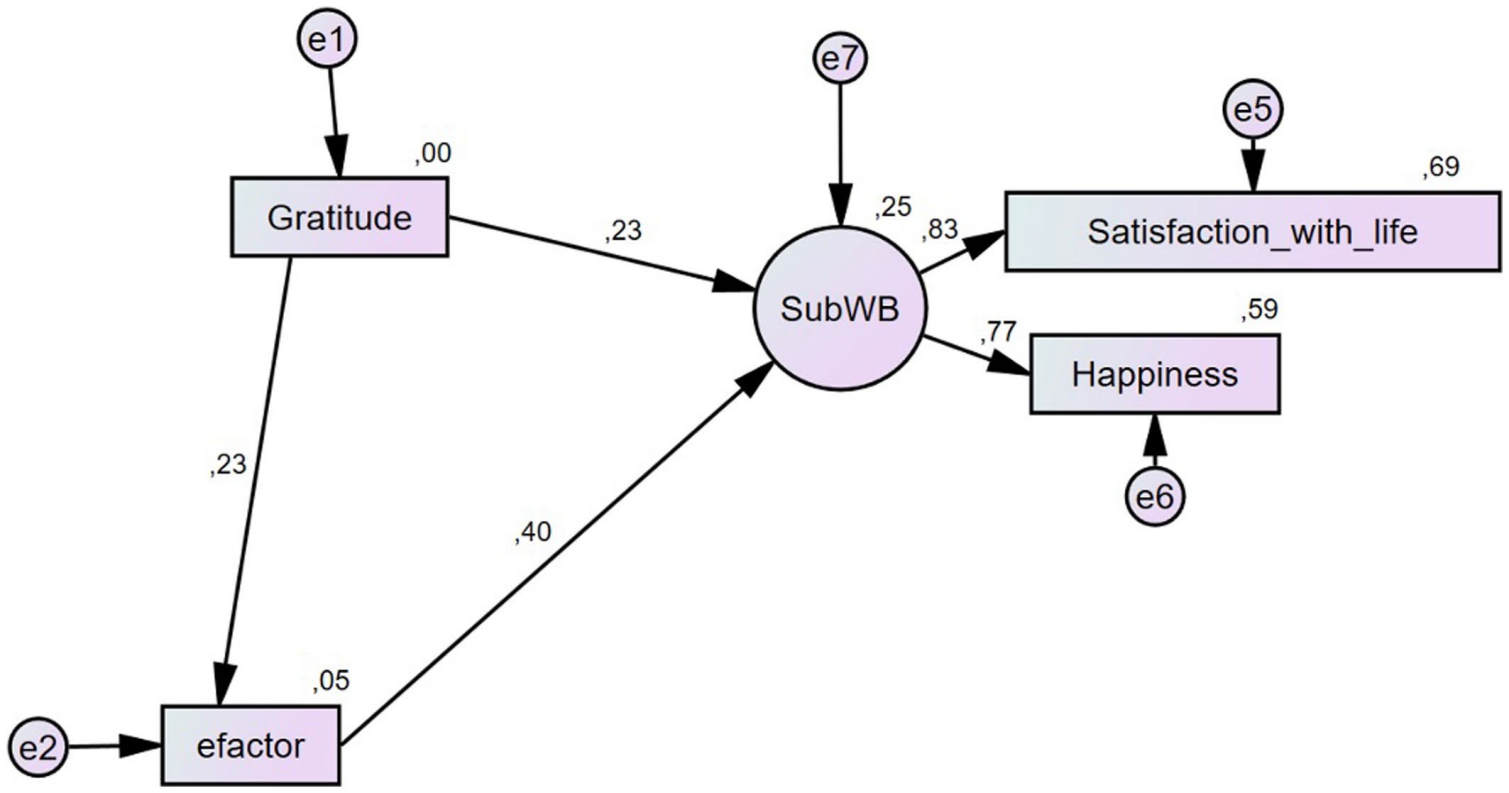
Sequential mediation model showing Model B, using gratitude as a mediator of the association between Bifactor and Subjective Well-being (SubWB or SWB).

As shown in Table 3, the unstandardized direct effects indicated that there were positive significant relationships among all variables, considering Model B (see Figure 2) as the one selected. Gratitude showed a low significant correlation with SWB (*β* = 0.376, *p* < 0.001) as well as with the *e*-factor (*β* = 0.218, *p* < 0.001). Indeed, it is the *e*-factor which had a higher correlation with SWB (*β* = 0.709, *p* < 0.001) even though it was conceived as a mediator. This information allows us to confirm the second hypothesis (H2), in which there was a significant correlation between gratitude and SWB; nonetheless, the relation is low which means that there are other factors not considered yet that may play a significant role in between.

**Table 3:** Sequential mediation model.

Measures	*β*	*SE*	ρ
Gratitude on *e*-factor	0.218	0.045	<0.001
Gratitude on Subjective Well-being	0.376	0.088	<0.001
*e*-factor on Subjective Well-being	0.709	0.097	<0.001
Subjective Well-being on Satisfaction with life	1.000		
Subjective Well-being on Happiness	0.792	0.084	<0.001

The mediated effects were then analyzed (see Table 4). We found significant overall effects among the variables; specifically, the mediating effect of the *e-*factor between gratitude and SWB was *β = 0*.155 (*p* < 0.001) but this effect was indeed small. Now, the mediating effect of the *e*-factor between gratitude and happiness was slightly higher (*β = 0*.420, *p* < 0.001), the same as what happened with satisfaction with life (*β = 0*.531, *p* < 0.001). This means that gratitude and *e*-factor explained 53% of the variance in satisfaction with life and 42% of the variance in happiness; gratitude explained 22% of the variance in *e*-factor. The findings show that the *e*-factor mediates the relationship between gratitude and SWB, having more effect on happiness and satisfaction with life, which confirms our third hypothesis (H3).

**Table 4:** Indirect effects and 95% confidence intervals (CI) for the model.

Relationships	Direct effect	Indirect effect	95% IC		*Two-tailed p-value*
			Lower	Upper	
Gratitude → *e*-factor	0.218	–	–	–	–
Gratitude → *e*-factor → Subjective Well-being	0.376	0.155	0.077	0.262	< 0.001
Gratitude → e-factor → Subjective Well-being → Happiness	0.000	0.420	0.243	0.586	< 0.001
Gratitude → *e*-factor → Subjective Well-being → Satisfaction with life	0.000	0.531	0.293	0.752	< 0.001
*e*-factor → Subjective Well-being	0.709	–	–	–	–
*e*-factor → Subjective Well-being → Happiness	0.000	0.562	0.387	0.751	< 0.001
*e*-factor → Subjective Well-being → Satisfaction with life	0.000	0.709	0.517	0.903	< 0.001
Subjective Well-being → Happiness	0.792	–	–	–	–
Subjective Well-being → Satisfaction with life	1.000	–	–	–	–

Unstandardized coefficients reported. Bootstrap sample = 5,000 replacement.

## 5 Discussion

Gratitude has been repeatedly identified by research as a factor that may enhance flexible thinking, positive emotions and effecting coping (for example, Fredrickson et al., 2003; Fredrickson, 2004; Adler and Fagley, 2005). Gratitude, at the same time, seems to be linked to other constructs like EI or emotional abilities (Wilks et al., 2014).

Additionally, many studies also showed that gratitude has a strong connection with well-being and happiness (Scurtu-Tura et al., 2024). Watkins et al. (2001), showed that those individuals who exhibited higher levels of gratitude as a trait, also showed higher levels of life satisfaction, a more important feeling of subjective well-being, and more positive emotions than those individuals with lower levels of gratefulness. Taking both facts together, Lin and Yeh (2014b) found that grateful individuals tend to employ active problem-focused coping and active emotion-focused coping more often, which facilitates their well-being.

The present study aimed at analyzing the relationship between gratitude and SWB in a group of Spanish undergraduate students, also exploring the effect of EI as a possible mediator, using the *e*-factor and its facets. We provided evidence of the relations among gratitude, trait EI and SWB, not considering EI facets because of their poor model fit. The results of the path analyses revealed that there was a mediation between gratitude and satisfaction with life and happiness, indicating that gratitude can significantly promote EI. At the same time, gratitude also predicted higher levels of SWB, but in less proportion. These findings suggest that greater EI might be the mechanism through which gratitude contributes to satisfaction with life and happiness (Watkins et al., 2001; Chang et al., 2022).

In order to explore the relation among these variables, this study comprised different steps. First of all, we analyzed the relationship between gratitude and SWB indicators (happiness and life satisfaction). Congruent with previous evidence, our results showed a positive association between both constructs. This comes together with the fact that thinking in a grateful manner is proven to enhance mood and positive affect (Watkins, 2014). Interestingly, prospective studies, which can give more information about stability over time, indicated that grateful people (understood as a trait gratitude) have the capacity to increase positive affect over time (Spangler et al., 2008). Thus, literature indicated that being grateful increases the frequency of positive affective experiences for the individual in a relatively stable way. Indeed, Kong et al. (2019a,b) demonstrated solid neural evidence that links trait gratitude to life satisfaction. Importantly, our results indicated that grateful people tend to have a more satisfactory life, but also more happiness, in other words, gratitude is beneficial for SWB. Moreover, this ties in with the broaden-and-build theory (Fredrickson and Losada, 2005; Kong et al., 2019a,b) and virtuous circle (Morgan et al., 2014; Watkins et al., 2014), and this association can demonstrate that grateful people tend to have a more satisfactory life and emotional well-being (Kashdan et al., 2006; Wood et al., 2008; Geng, 2018; Kong et al., 2019a,b).

The second goal of the present study was to examine the mediation role of the general EI (*e*-factor) and its facets as a potential mediator between gratitude and SWB. According to previous research (Blasco-Belled et al., 2019), we introduced the bifactor analysis in this study to explain general EI. This bifactor analysis showed better adjustment than other structures, the *e*-factor, which contained shared information that helped explain the general self-perceived ability to rationalize emotions. Additionally, the present study took into account three independent specific emotional facets that provide additional information beyond the *e*-factor (mood attention, emotional clarity, and mood repair). However, we were not able to separate each of the three emotional facets and examine their unique contribution to life satisfaction and happiness beyond the *e*-factor, as did in other researches (Blasco-Belled et al., 2019; Tejada-Gallardo et al., 2020). While the literature showed a positive association between EI and SWB (Di Fabio and Kenny, 2016; Kong et al., 2019a,b), in our results we found a positive relation between general EI (*e*-factor) and SWB.

Despite that, our results were consistent with previous literature, which showed that grateful people tend to focus their attention on appreciating the positive in life (Wood et al., 2010; Kong et al., 2019a,b), which can promote happiness and increase positive affect. In this sense, grateful people have the capacity to focus on positive emotions and neutralise the negative (Tugade and Fredrickson, 2002). In other words, grateful people seem to be more able to benefit from their broadened mindsets and to regulate their negative emotional experiences. This enhanced regulation skill, in turn, produces important benefits in the experienced happiness levels of the person, as well as in their SWB. Generally, this is consistent with previous literature, in which grateful people appeared to be particularly good at repairing their unpleasant moods and choosing adaptive coping strategies (Wood et al., 2008; Lambert et al., 2012).

Grateful people have a tendency to feel better with their life, have better expectations and have predisposition to certain emotional states (Ramzan and Rana, 2014). In our case, this is evident from the fact that trait EI shows an association with SWB, which takes into consideration both components cognitive rather than emotional. Koydemir et al. (2012) demonstrated that trait of EI is more significant to wellbeing than ability EI; and Gallagher and Vella-Brodrick (2008) demonstrated that EI is related to both components of SWB. Higher emotional intelligent people have the capacity to be more adaptive, to deal with difficult situations and hassles of everyday life more easily. Moreover, the *e*-factor of EI in the mediator role between gratitude and SWB, allows people to perceive, understand and manage their emotions hence enabling them to have better SWB. Not only that, but grateful people have the capacity to feel and experiment more positive emotions, and this can amplify and promote EI; as a result, it could increase people’s levels of SWB (Watkins, 2014; Sánchez-Álvarez et al., 2016). In other words, this leads to evaluating one’s life positively (life satisfaction and a more cognitive component) and experiencing relatively more positive than negative emotions (affect balance and subjective component) (Diener et al., 2003; Brülde, 2007; Blasco-Belled et al., 2019).

All in all, our results showed that gratitude seems to help create a mindset that improves EI, and, at the same time, EI enhances and facilitates SWB.

This study represents a first attempt to investigate general EI as underlying mechanisms of the relationship between gratitude and subjective well-being indicators (e.g., life satisfaction and happiness). The results of the mediating role of the *e*-factor EI between gratitude and subjective well-being indicators might further shed light on the complex relationships among these variables. However, additional studies should be conducted to confirm these findings.

In addition, these findings suggest that the cultivation of gratitude may work as an enhancer of SWB. It may also function as an active strategy to increase individuals’ resources according to the broaden-and-build theory and to focus in a virtuous circle, thus increasing their happiness and life satisfaction.

### 5.1 Limitations and theoretical contributions

Some limitations in the current study should be addressed. The first limitation is that the data relied exclusively on self-report measures. The use of multiple methods for evaluation may lower the influence of subjectivity. The second limitation is that this study was a cross-sectional design; a longitudinal and experimental study would provide additional insights into the relationships among gratitude, trait emotional intelligence, and subjective well-being indicators (e.g., life satisfaction and happiness).

Third, despite the fact that the sample was adequate in terms of number and representativeness, at a global level, our participants were majority females (79.5%). Thus, in future research, it would be interesting to use a more heterogeneous sample to confirm and generalize our results. Fourthly, given the study sample, the generalization of the results should be taken with caution. Last but not least, the study group was composed solely of university students, which limited the generalizability of the findings of the current study; cross-validating the model to other contexts and ages would be a first step to overcome this limitation.

Despite these limitations, the present study helped extend our insight into which mechanism of gratitude modulates subjective well-being. This study also provides preliminary evidence of the relations between gratitude and subjective well-being. These findings may provide valuable guidance for how to implement psychological interventions aimed at enhancing subjective well-being thorough EI. Moreover, these findings support the broaden-and-build theory (Tugade and Fredrickson, 2002) and amplify the importance of using positive emotions on the construction of EI. According to this theory and the virtuous circle theory (Watkins, 2014; Watkins et al., 2014), grateful people have the capacity to focus their attention on the positive emotions, which improves their subjective well-being. The study of the role of the personality variables between gratitude and EI and SWB is suggested as another possible line of study.

However, it is important to consider different strategies, such as train-teach-coach to develop how people can effectively utilize their knowledge of positive emotions in opportune moments to optimize their well-being and their personal and social growth. Scholars have demonstrated that an emotionally intelligent person can fully appreciate the advantages of positive emotions. Further studies should focus on alternative mechanisms by utilising experimental designs to enrich the path models supported by the present study.

## Data availability statement

The datasets presented in this study can be found in online repositories. The names of the repository/repositories and accession number(s) can be found below: https://osf.io/zgbrp/?view_only=53e9b3bd48624caf86cf1956a800dd6f.

## Ethics statement

Ethical review and approval was not required for the study on human participants in accordance with the local legislation and institutional requirements. Written informed consent from the [patients/ participants or patients/participants legal guardian/next of kin] was not required to participate in this study in accordance with the national legislation and the institutional requirements.

## Author contributions

CT-N: Writing – original draft, Writing – review & editing. AR-M: Writing – original draft, Writing – review & editing. CQ-P: Writing – original draft, Writing – review & editing.
